# Upregulation of MTA1 in Colon Cancer Drives A CD8^+^ T Cell-Rich But Classical Macrophage-Lacking Immunosuppressive Tumor Microenvironment

**DOI:** 10.3389/fonc.2022.825783

**Published:** 2022-03-08

**Authors:** Yantong Zhou, Peng Nan, Chunxiao Li, Hongnan Mo, Ying Zhang, Haijuan Wang, Dongkui Xu, Fei Ma, Haili Qian

**Affiliations:** ^1^ State Key Laboratory of Molecular Oncology, National Cancer Center/National Clinical Research Center for Cancer/Cancer Hospital, Chinese Academy of Medical Sciences and Peking Union Medical College, Beijing, China; ^2^ Department of Medical Oncology, National Cancer Center/National Clinical Research Center for Cancer/Cancer Hospital, Chinese Academy of Medical Sciences and Peking Union Medical College, Beijing, China; ^3^ Department of Gynecological Minimal Invasive Center, Beijing Obstetrics and Gynecology Hospital, Capital Medical University, Beijing Maternal and Child Health Care Hospital, Beijing, China; ^4^ The Editorial Office of Infectious Diseases & Immunity, Chinese Medical Journals Publishing House Co., Ltd, Beijing, China; ^5^ Department of VIP, National Cancer Center/National Clinical Research Center for Cancer/Cancer Hospital, Chinese Academy of Medical Sciences and Peking Union Medical College, Beijing, China

**Keywords:** MTA1, immune infiltration, tumor associated macrophage (TAM), tumor microenvironment, colorectal cancer

## Abstract

**Background:**

The MTA1 protein encoded by metastasis-associated protein 1 (MTA1) is a key component of the ATP-dependent nucleosome remodeling and deacetylase (NuRD) complex, which is widely upregulated in cancers. MTA1 extensively affects downstream gene expression by participating in chromatin remodeling. Although it was defined as a metastasis-associated gene in first reports and metastasis is a process prominently affected by the tumor microenvironment, whether it affects the microenvironment has not been investigated. In our study, we elucidated the regulatory effect of MTA1 on tumor-associated macrophages (TAMs) and how this regulation affects the antitumor effect of cytotoxic T lymphocytes (CTLs) in the tumor microenvironment of colorectal cancer.

**Methods:**

We detected the cytokines affected by MTA1 expression *via* a cytokine antibody array in control HCT116 cells and HCT116 cells overexpressing MTA1. Multiplex IHC staining was conducted on a colorectal cancer tissue array from our cancer cohort. Flow cytometry (FCM) was performed to explore the polarization of macrophages in the coculture system and the antitumor killing effect of CTLs in the coculture system. Bioinformatics analysis was conducted to analyze the Cancer Genome Atlas (TCGA) colorectal cancer cohort and single-cell RNA-seq data to assess the immune infiltration status of the TCGA colorectal cancer cohort and the functions of myeloid cells.

**Results:**

MTA1 upregulation in colorectal cancer was found to drive an immunosuppressive tumor microenvironment. In the tumor microenvironment of MTA1-upregulated colorectal cancer, although CD8^+^ T cells were significantly enriched, macrophages were significantly decreased, which impaired the CTL effect of the CD8^+^ T cells on tumor cells. Moreover, upregulated MTA1 in tumor cells significantly induced infiltrated macrophages into tumor-associated macrophage phenotypes and further weakened the cytotoxic effect of CD8^+^ T cells.

**Conclusion:**

Upregulation of MTA1 in colorectal cancer drives an immunosuppressive tumor microenvironment by decreasing the microphages from the tumor and inducing the residual macrophages into tumor-associated microphage phenotypes to block the activation of the killing CTL, which contributes to cancer progression.

## Introduction

With the development of cancer research, the concept of the tumor microenvironment and its importance in tumor progression and tumor treatment is increasingly being recognized (1). In the process of cancer development, all types of cells and products in the tumor microenvironment influence tumorigenesis and tumor progression. The composition and functional status of immune cells in the tumor micro-environment are the focus of studies in both fields of basic immunotherapy research and clinical application. Accordingly, immunosignature-based stratification of patients to optimize the treatment response is becoming central to cancer immunotherapy. Preliminarily, the tumor immune microenvironment has been defined as cold versus hot tumors based on their CTL infiltration. An immunologically cold microenvironment is primarily associated with the exclusion of cytotoxic CD8^+^ T cells in tumors (2). However, hot tumors rich in infiltrated CD8^+^ T cells may still fail to respond to the immune checkpoint blockade (ICB) if these cells are dysfunctional or become exhausted (3). An inflamed but still immunosuppressive microenvironment can be potentially fueled by various myeloid cells, particularly tumor-associated macrophages (TAMs) (4, 5). Unfortunately, drivers of these different immunomodulatory scenarios are not well defined. Therefore, it is critical to define the mechanisms of immune aberrance in terms of not only the accounts but also the functions of immune cells in the tumor microenvironment to design further optimized cancer immunotherapy strategies.

MTA1, as an important component of the nucleosome-remodeling complex (6), widely participates in gene expression regulation. MTA1 was first discovered as a gene related to tumor metastasis in breast cancer, and more of its biological function was revealed in subsequent studies. It widely regulates the biological behaviors of tumor cells. In our investigation, we found clues about MTA1 involved in the regulation of the immune state in the tumor microenvironment and contributed to an important regulatory link between macrophages and CD8^+^ T cells, which has been known as a core process in tumor microenvironment regulation but is still poorly understood.

In the current study, we sought to investigate the influence of MTA1 overexpression on the antitumor immune response. Based on the clue that MTA1 has been associated with inflammation in colorectal cancer, we primarily tried to explore how overexpressed MTA1 influences the immune infiltration states and immune response in the tumor microenvironment of a TCGA colorectal cancer cohort. Then, we analyzed the function of macrophages modulated by MTA1-overexpressing colorectal cancer cells. To determine their relevance to overexpressed MTA1, a multiplex immunohistochemistry (m-IHC) panel consisting of surface and intracellular markers was designed to perform on clinical colorectal cancer samples. Using this panel, we described predominant TAM populations, T cell populations and B cell populations characterized by specific markers on individual cells. Finally, we performed a cytotoxicity assay to explore the influence of MTA1 overexpression on the antitumor immune response. Based on the above experimental results, we set out to illustrate how MTA1 affected the interactions between cancer cells, macrophages, and cytotoxic T lymphocytes to contribute to an immunosuppressive microenvironment.

## Materials and Methods

### Cell Culture

Mouse colorectal cancer CT26 cells and human colorectal cancer HCT116 cells were used as models to express high and low MTA1. RAW264.7 cells were used as an *in vitro* model of murine macrophages. Mouse spleen lymphocytes were isolated to perform an *in vitro* cytotoxicity assay for T cell functions. HCT116 cells and RAW264.7 cells were cultured in Dulbecco’s modified Eagle’s medium (DMEM; CELL Technologies) with 100 μg/ml penicillin/streptomycin supplemented with 10% fetal bovine serum (FBS; Gibco). CT26 cells were cultured in Roswell Park Memorial Institute (RPMI-1640; CELL Technologies) supplemented with 100 μg/ml penicillin/streptomycin and 10% fetal bovine serum (FBS; Gibco).

### Study Cohort

The Molecular Analysis of Colorectal Cancer (CRC) cohort consisted of 180 colorectal cancer patients from the Cancer Hospital, Chinese Academy of Medical Sciences. Written informed consent was obtained from all patients prior to sample collection. All procedures were ethically approved by The Independent Ethics Committee of Cancer Hospital, Chinese Academy of Medical Sciences.

### Multiplex IHC Staining Protocol

An Opal 7-color kit (PANOVUE) was used for multiplex IHC. Four micrometers of FFPE sections were dewaxed and rehydrated. In the first round, antigen was retrieved with a microwave oven (EDTA pH 8.0) at 70% power for 15 min (Galanz, G80F23CN2P-BM1). Slides were cooled to room temperature (RT) and washed with PBST/0.5% Tween (3 times, 5 min). Slides were washed and blocked with goat serum blocking buffer (zsbio) for 10 min. Primary antibody was incubated at RT for 1 h or at 4 °C overnight. Slides were washed, and an HRP-conjugated secondary antibody was incubated at RT for 10 min. TSA dye (1:100) was applied for 10 min after washing. These procedures were repeated five more times using the following antibodies: CD8a (Cell Signaling Technology, 1:500), CD4 (zsbio, ZA-0519, 1:1), MTA1 (Abcam, AB71153, 1:1,000), CD20 (Cell Signaling Technology, #3958, 1:200), CD163 (Abcam, ab182422, 1:200), CD206 (Abcam, ab64693, 1:500), CD86 (Abcam, ab269587, 1:200), CD68 (Abcam, ab955, 1:1,000), and PD-L1 (Cell Signaling Technology, E1L3N, 1:200).

### Multiplex IHC Imaging and Analysis

Stained tissue arrays were imaged using a PerkinElmer Vectra Polaris microscope. Whole slide scans were performed using the ×20 objective lens. ROIs were selected in Phenochart (PerkinElmer) based on the images previously captured on the whole slide scan. For selecting ROIs, 2 × 2 core stamps were used. The image analysis was performed in inForm2.2.4 (PerkinElmer). Tissue component segmentation was conducted to label tumor tissue (α-SMA) and stromal tissue (α-SMA+) regions. Cell phenotyping was performed using previously reported markers (CD8, CD4, CD20, CD163, CD206, CD86, CD68). The density of cells in each tissue type was calculated by normalizing the cell counts from all images by the total area.

### Quantification of MTA1-Associated Secretion

HCT116 cells were incubated in DMEM with 10% FBS before collection, and MTA1-associated secretion was estimated by cytokine antibody array (CAA) following the manufacturer’s instructions.

#### Coculture System

CT26–MTA1-knockdown, -overexpressing or control cells (2 × 10^5^) were seeded in 6-well culture dishes for 48 h. RAW264.7 cells were then seeded together with these CT26 cell populations at a 1:2 ratio in RMPI medium with 100 μg/ml penicillin/streptomycin (Gibco) containing 10% heat-inactivated FBS (Gibco) and 200 nM glutamine. After 48 h, cells were trypsinized and then underwent flow cytometry assay or qRT–PCR assay.

### RNA Extraction and qRT–PCR

Total RNA was extracted and purified from cell pellets using an RNeasy Mini-Kit (Qiagen) following the manufacturer’s instructions. The RNA concentration was determined by a NanoDrop Spectrophotometer ND-1000 (NanoDrop Biotechnologies). Total RNA (1–2 μg) was retrotranscribed into cDNA using a High-Capacity cDNA Reverse Transcriptase Kit (Thermo Fisher Scientific) according to the manufacturer’s protocol. Then, 20 ng of the total cDNA was subjected to qRT–PCR (at an annealing temperature of 60°C) using Power SYBR Green PCR Master Mix (TAKARA). Assays were performed in triplicate on a QuantStudio 5 Flex Real-Time PCR System (Applied Biosystems). The forward and reverse primer sequences were as follows: Arg-1(forward: 5′- CCACAGTCTGGCAGTTGGAAG; reverse: 5′- GGTTGTCAGGGGAGTGTTGATG); Ym-1 (forward: 5′- CCCTTCTCATCTGCATCTCC; reverse: 5′- AGTAGCAGTCATCCCAGCA); IL-6 (forward: 5′- GGACCAAGACCATCCAATTC; reverse: 5′- ACCACAGTGAGGAATGTCCA); Saa3 (forward: 5′- GCCTGGGCTGCTAAAGTCAT; reverse: 5′- TGCTCCATGTCCCGTGAAC); Gapdh (forward: 5′- AATGTGTCCGTCGTGGATCTGA; reverse: 5′- GATGCCTGCTTCACCACCTTCT); TNF-α (forward: 5′- AGCCCACGTCGTAGCAAACCAC; reverse: 5′- AGGTACAACCCATCGGCTGGCA); CD206 (forward: 5′- GGCAGGATCTTGGCAACCTAGTA; reverse: 5′- GTTTGGATCGGCACACAAAGTC); iNOS (forward: 5′- TCCTGGACATTACGACCCCT; reverse: 5′- CTCTGAGGGCTGACACAAGG);

### T Cell Cytotoxicity Assay

CT26–MTA1-knockdown, -overexpressing or control cells (2 × 10^5^) were seeded in 24-well culture dishes for 48 h. RAW264.7 cells were then seeded together with these CT26 cell populations at a 1:2 ratio in RPMI medium with 100 μg ml^−1^ penicillin/streptomycin (Gibco) containing 10% heat-inactivated FBS (Gibco) and 200 nM glutamine. After 48 h, 4 × 10^5^ CD3- and IL-2-activated T effector cells were added to the culture for an additional 6 h. Cells were trypsinized and stained with Annexin-V, as described below, and analyzed by flow cytometry. Before staining with annexin V, CD45 (Invitrogen, 12-0451-81, 0.015 μg/100 μl) was stained to identify macrophages and lymphocytes from the tumor cells. FSC-A and SSC-A, representing volume and granularity, were used to distinguish macrophages from lymphocytes. In addition, CD11b (Invitrogen, 25-0112-81, 0.06 μg/100 μl) was used to identify macrophages in the coculture system.

### Immune Profiling by Flow Cytometry

For the coculture experiment for cell surface staining, 1 × 10^6^ cells were incubated with anti-Fc receptor blocking antibody and stained with the indicated antibodies in PBS, 2% bovine serum albumin and 5 mM EDTA for 30 min on ice. All flow cytometry was performed on a BD flow cytometer (BD FACS ARIA II). Analysis of flow cytometry data was performed using FlowJo_v10.6.2. Flow cytometry antibodies were used as follows: CD45 (Invitrogen, 12-0451-81, 0.015 μg/100 μl), CD11b (Invitrogen, 25-0112-81, 0.06 μg/100 μl), PD-L1 (Biolegend, 124307, 1:50), PD-1 (Biolegend, 114117, 1:50), Ifng (Biolegend, 505809, 1:50), Tigit (Biolegend, 142105, 1:50), CD206 (Abcam, ab64693, 1 μg/ml), CD86 (Abcam, ab269587, 1:50), and CD163 (Abcam, ab182422, 1:60). Cells were discriminated using the following combinations of cell markers after gating on single cells (discriminated by forward scatter area and forward scatter width). CD45 was labeled to select macrophages and T cells. FSC-A, SSC-A and CD11b were labeled to distinguish T cells and macrophages. CD86, CD163 and CD206 were labeled to detect the polarization phenotypes of macrophages.

### Bioinformatics Analysis: GSVA and Score Generation

For gene set enrichment analysis (GSEA), we used the Broad Institute Molecular Signatures Database (MSigDB) (https://www.gsea-msigdb.org/gsea/msigdb) to obtain gene annotations of the pathways of interest as the background. In addition, we mapped the collected genes to the background. The R package clusterProfiler (Version 3.14.3) was used for enrichment analysis to obtain the results of gene set enrichment. The minimum gene set included 5 genes, the maximum gene set included 5,000 genes, and a P-value <0.05 and FDR <0.01 were considered statistically significant.

For differential expression analysis, we used the R package limma (version 3.40.6) to obtain differentially expressed genes between groups. For gene set variation analysis (GSVA), we used the R package GSVA (version 1.40.1) (7). Based on RNA expression profiling, we used the Broad Institute Molecular Signatures Database (MSigDB) (https://www.gsea-msigdb.org/gsea/msigdb) to obtain gene annotations of the pathways of interest as the background to score pathways and molecular mechanisms in each sample.

For survival analysis, we used the R package Survival after integrating overall survival time, survival state and expression data of MTA1. In addition, the significance was assessed by COX (8).

Single-cell RNA-seq data were extracted from the Gene Expression Omnibus (accession code GSE132465), comprising 63,687 cells from 23 patients with colorectal cancer, using the provided normalization and cell labels (9). Bulk RNA-seq data of the TCGA colorectal cancer cohort were downloaded from the cBioPortal (http://www.cbioportal.org/).

### Statistical Analysis

Two-sided *p*-values of less than 0.05 were considered statistically significant (ns, not significant; *p <0.05; **p <0.01; ***p <0.001; ****p <0.0001). All statistical analyses were performed in GraphPad 8.3.

## Results

### The High Level of MTA1 Expression in the TCGA Colorectal Cancer Cohort was Significantly Associated With the Immunosuppressive Signature

**Figure 1:**
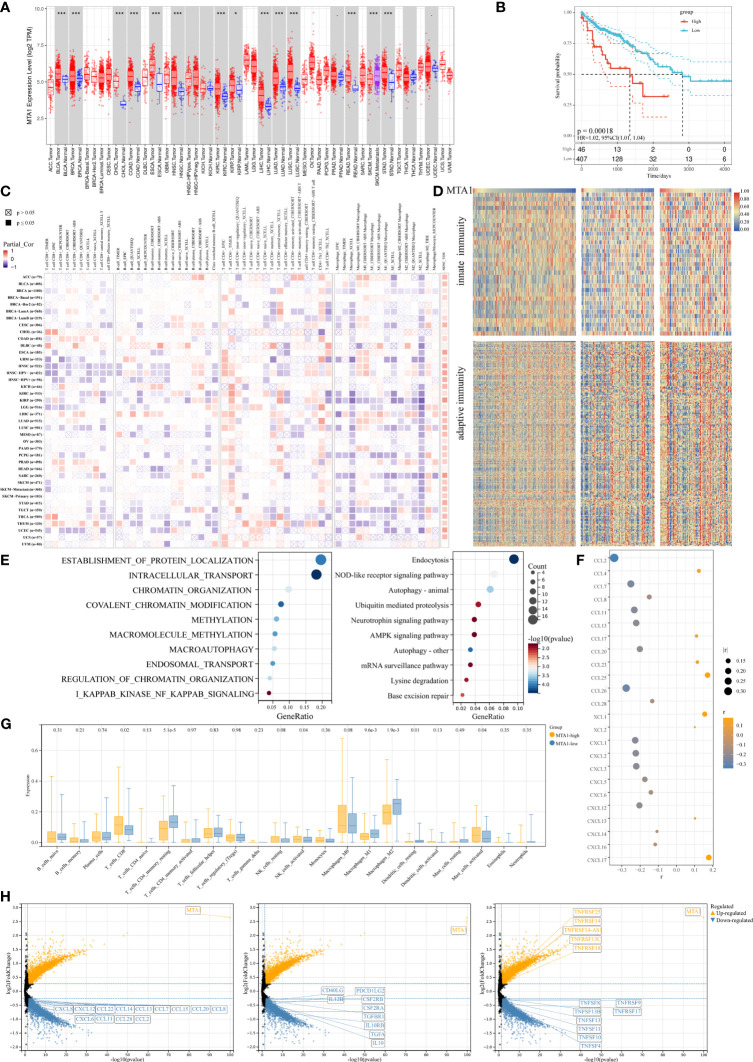
MTA1-driven immunosuppressive tumor microenvironment in colorectal cancer. **(A)** Pan-cancer expressional characterization of MTA1 in tumor and normal tissues from the TCGA database. **(B)** Kaplan–Meier curves of overall survival of patients with MTA1-high and MTA1-low tumors. The MTA1-high group included patients whose tumors expressed MTA1 mRNA at the level of the upper 25%. The MTA1-low group included patients whose tumors expressed MTA1 mRNA at the level of the lower bottom 25%. COAD and colon adenocarcinoma were defined in the TCGA dataset. P-values correspond to two-sided log-rank analyses. **(C)** Immune infiltration based on bulk RNA-seq transcriptome data from the TCGA database assessed by different algorithms. **(D)** Heatmap representing the correlation between MTA1 expression level and enrichment score defined by GSVA of signaling cascades from MSigDB related to immunomodulation in the TCGA colon adenocarcinoma cohort. **(E)** Gene set enrichment of the top 500 MTA1 coexpression genes (p-adjust <0.05; cluster Profiler). **(F)** Pearson correlation between the expression of MTA1 and chemokines in colon cancer cell lines in the CCLE database (p-value <0.05). **(G)** Enrichments of the indicated immune cell populations calculated *via* the Cibersortx algorithm in MTA1-high versus MTA1-low populations (low: n = 92; high: n = 87; unpaired two-tailed t test). **(H)** Differentially expressed gene (DEG) in MTA1-high versus MTA1-low populations (low: n = 128; high: n = 128; limma). The meaning of symbols was provided in Statistical Analysis.

MTA1 is generally overexpressed in tumors, and also in colorectal cancer (**Figure 1A**), and the MTA1 expression level is negatively correlated with the overall survival of the patients in the colorectal cancer cohort (**Figure 1B**). To explore the effect of MTA1 expression on colorectal cancer immune cell infiltration in the tumor microenvironment, we used the TIMER 2.0 (10) database to evaluate the correlation between the MTA1 expression level and immune cell infiltration score in the colorectal cancer cohort of the TCGA (The Cancer Genome Atlas, TCGA) database. Among the results derived from various immune infiltration evaluation algorithms, the expression level of MTA1 in colorectal cancer was significantly positively correlated with the level of CD8^+^ T cell infiltration and negatively correlated with macrophage infiltration (**Figure 1C**). To further evaluate the influence of the MTA1 expression level on the status of the immune response in colorectal cancer, we used ssGSVA to evaluate the innate immunomodulation enrichment score and adaptive immunomodulation enrichment score of each colorectal cancer patient cohort in the TCGA database. The results showed that colorectal cancer patients with high levels of MTA1 expression tended to be enriched with lower immunomodulation enrichment scores, which reflects an immunosuppressive status in their tumor tissues (**Figure 1D**). Gene set enrichment analysis of the top 500 MTA1 coexpression genes analyzed by the cluster profiler revealed immune biological processes at the top of the list, suggesting that MTA1 may be immune signature-related in colorectal cancer. In the results derived from the Gene Ontology (GO, **Figure 1E**, left) database and the Kyoto Encyclopedia of Genes and Genomes (KEGG, **Figure 1E**, right) database, the MTA1 expression level may significantly affect the NF-κB pathway and the NOD-like receptor signaling pathway (**Figure 1E**). The immune cell infiltration status and immune response status were based on the recruitment and activation of immune cells. In the tumor microenvironment, the recruitment and activation of immune cells rely mostly on cytokines and chemokines. We then analyzed the correlation between the levels of chemokines and MTA1 in colorectal cancer cell lines in the Cancer Cell Line Encyclopedia (CCLE) database, and there was a significant negative correlation between the levels of MTA1 and the panel of chemokines. Most of the chemokines were downregulated with MTA1 overexpression in colorectal cancer cell lines (**Figure 1F**).

We then performed a more detailed deconvolution of 22 immune cell types with CIBERSORTx (11), which showed significantly enriched scores for CD8^+^ T cells, memory resting CD4^+^ T cells, activated NK cells, DC cells, mast cells, and regulatory T cells (Treg cells) and, more notably, for macrophages in the MTA1-high group versus the MTA1-low group of colorectal cancers (**Figure 1G**). Among the significantly affected immune cells, direct tumor killers, CD8^+^ T cells and NK cells were enhanced with MTA1 upregulation. The immune cells supporting the effect of cytotoxic cells were generally decreased with MTA1 upregulation.

The differentially expressed gene sets between patient populations within the top and bottom 25th percentiles of the MTA1 expression level mainly fell in the family of chemokines (**Figure 1H**, left), common immunostimulators (**Figure 1H**, middle), and immunosuppressors (**Figure 1H**, right), as shown. Thus, we found that in MTA1-upregulated colorectal cancer, immune factors were generally downregulated. Altogether, these above data suggest that the expression of MTA1 significantly affects the immune status in colorectal cancers.

### Colorectal Cancer Cells Expressing High Levels of MTA1 Downregulate the Expression of Cytokines Recruiting Monocytes and Macrophages

We found that chemokines were mostly downregulated in the MTA1-high group, among which CCL2 was one of the most prominent chemokines. The results derived from CCLE database analysis also displayed a significant difference in the expression of CCL2 between tumor cells with high or low MTA1 levels. Solid tumors are continuously seeded by blood monocytes to sustain large intratumoral macrophage populations (12), and monocyte recruitment to the tumor bed is dependent on the CCL2–CCR2 axis (13–16). The downregulation of CCL2 in MTA1-overexpressing colorectal cancer impaired the recruitment of monocytes and finally formed macrophages with a relatively decreased tumor microenvironment.

**Figure 2:**
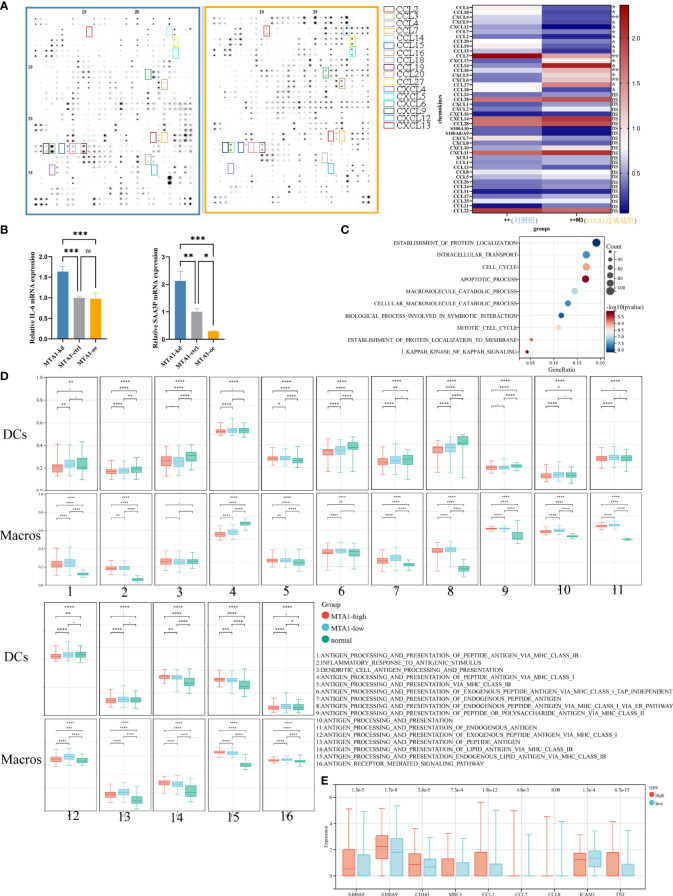
MTA1-driven immunosuppressive secretome and educated myeloid cells in colorectal cancer. **(A)** Expressional characterization of chemokines in MTA1-overexpressing and control HCT116 human colorectal cancer cells. **(B)** qRT–PCR detected the expression of cytokines, the inflammation stimulators IL-6 and SAA3P, in MTA1-knockdown, MTA1-overexpressing and control CT26 mouse colorectal cancer cells. **(C)** Gene set enrichment analysis of differentially expressed genes between MTA1-knockdown and control HCT116 cells. **(D)** Antigen presentation-associated gene set enrichment scores of dendritic cells (DCs) and macrophages (Macros) in the MTA1-high tumor group, MTA1-low tumor group and normal tissue group. The expression profiles of single DCs or macrophages were used to perform GSVA. **(E)** Tumor-associated macrophage expressional features of macrophages in the MTA1-high tumor group versus the MTA1-low tumor group. The expression profiles of single macrophages were used to perform differential analysis. The meaning of symbols was provided in Statistical Analysis.

To confirm the results derived from the TCGA COAD cohort, we performed a cytokine antibody array to detect cytokine secretion in HCT116 human colon cancer cells of the control group and MTA1-overexpressing group (**Figure 2A**). Consistent with the results derived from bioinformatic analysis, CCL2, CCL7, CCL20, and CXCL12 were all downregulated in MTA1-overexpressing cells. In particular, the downregulation of CCL2 was significantly associated with the absence of macrophages in the MTA1-high tumor group. In addition to the downregulation of cytokines, the inflammatory stimulators IL-6 and SAA3P were also downregulated in MTA1-overexpressing CT26 mouse colon cancer cells (**Figure 2B**). Gene set enrichment analysis was performed to analyze the differentially expressed genes in the MTA1-knockdown HCT116 cells versus the control HCT116 cells, and the NF-κB pathway was also enriched, as shown by the TCGA results (**Figure 2C**).

To illustrate the effect of MTA1 expression in cancer cells on the function of macrophages, we analyzed single-cell RNA-seq data from the GEO database. In this analysis, tumor cells, DCs and macrophages were the main cell types of concern. We aimed to explore how MTA1-overexpressing tumors affected the function of DCs and macrophages; therefore, we divided the samples into the MTA1-high group and the MTA1-low group based on the MTA1 expression level in tumor cells. Then, we assessed the enrichment score of antigen presentation-associated pathways in DCs and macrophages by the GSVA algorithm. Finally, we compared the enrichment score of antigen presentation-associated pathways in the MTA1-high group with the MTA1-low group. The results revealed a significant downregulation of antigen presentation-associated pathways in the MTA1-high group (**Figure 2D**). In addition, we found that markers of M2-like markers for tumor-associated macrophages were significantly upregulated in macrophages in the MTA1-high samples (**Figure 2E**) (17, 18).

The preliminary analysis of colon cancer cells and single-cell RNA-seq data revealed that high-level MTA1 tends to transform the function and quantity of macrophages into an immunosuppressive state.

### Higher MTA1 Expression in Colorectal Cancer was Linked to Lower Macrophage Density and Higher Density of CD8^+^ T Populations

**Figure 3:**
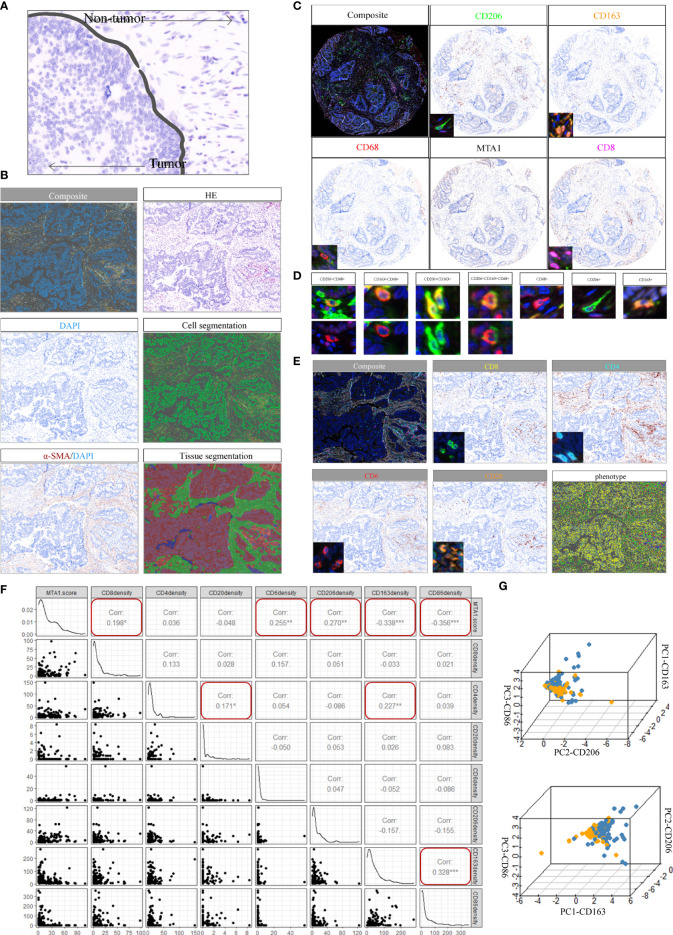
Spectrum landscape of the MTA1-driven tumor microenvironment. **(A)** Regions of interest (ROIs): tumor tissue and nontumor tissue. **(B)** Composite image (unfiltered image), H&E, single-stained DAPI, cell segmentation of single cells, single-stained α-SMA, and tissue-component segmentation of the same region. **(C)** Representative composite and single-stained IHC images of multiplex IHC panel No. 1. **(D)** Major TAM populations. Positivity (+) of corresponding markers and relative intensity between populations are indicated. **(E)** Representative composite and single-stained IHC images of multiplex IHC panel No. 2. **(F)** Pearson correlation between MTA1 score and immune cell infiltration density in colorectal cancer patients. (*p-value <0.05, **p-value <0.01, ***p-value <0.001). **(G)** 3D plots showing the intensities of TAM populations per group.

To investigate the immune landscape within colorectal cancer, tissue arrays from 180 patients were prepared from paraffin-embedded (FFPE) tumor samples (**Supplementary Table 1**; see *Materials and Methods* section). A set of H&E-stained tissue sections was reviewed by an anatomical pathologist (CM) to identify tumor (T) and nontumor normal (N) regions, which we referred to as regions of interest (ROIs) (**Figure 3A**; see *Materials and Methods*). The serially sectioned tissue arrays were stained with the m-IHC panel (**Figure 3B**, HE) to distinguish ROIs. A supervised image analysis system (inForm33) was used to identify every single cell by the positive index of DAPI (**Figure 3B**) and to segment each image into tumor areas (α-SMA−), nontumor (α-SMA+) areas and blank areas (**Figure 3B**). In addition, cell phenotyping data were obtained based on the patterns of marker expression (**Figures 3C–E**).

Macrophages in the tumor microenvironment are roughly classified into three populations: tumor-associated macrophages (TAMs) derived from recruited monocytes, tissue-resident macrophages and myeloid-derived suppressor cells (MDSCs). TAMs are the most abundant population in the tumor microenvironment. The polarization status of macrophages is roughly classified as classically activated M1 and alternatively activated M2. Classically activated M1 subtypes are recognized as proinflammatory and express markers such as MHC II, CD68, CD80, CD86 and INOS (19). Alternatively, activated M2 subtypes are recognized as anti-inflammatory and express markers, such as CD206, CD163 and Arg-1. The markers expressed by subtypes displayed differential metabolizing status. CD206 is a marker of glutamine metabolism (20), CD163 is a marker of iron metabolism (21), and ARG1 is associated with tumor-derived lactic acid (22). Therefore, three macrophage markers were analyzed at the single-cell level, and three typical populations were characterized and validated (**Figure 3D**). CD68 positivity was used as the criterion to identify macrophages (**Figure 3D**). Macrophages were further subdivided based on the positivity and relative intensity of other markers. M1-like TAM populations were identified by the CD163^−^/CD206^−^/CD86^+^ phenotype. M2-like TAM populations were identified by the presence of CD163^+^ or CD206^+^ (23).

We also stained CD8, CD4 and CD20 to monitor the infiltration of CD8^+^ T cells, CD4^+^ T cells and B cells (**Figure 3E**). The results showed that MTA1 expression was significantly negatively correlated with the infiltration density of CD68^+^/CD86^+^ macrophages, while it was significantly positively correlated with the infiltration of CD8^+^ T cells and CD68^+^/CD206^+^ macrophages. In addition, we found a significant correlation between CD4^+^ T cell infiltration density and CD20^+^ B cell infiltration density in the clinical colorectal cancer cohort (**Figure 3F**). Although the infiltration of CD4^+^ T cells and CD20^+^B cells was not significantly associated with the expression of MTA1, but the significant correlation and intercellular interaction between CD4^+^ T and B cells have been mentioned in numerous studies. B cells was generally recognized as a positive factor for immunotherapy response, especially B cells which are localized in lymphoid follicles of tertiary lymphoid structures (TLS) that increased percentages of tumor antigen-specific B cells and induced antitumor responses of T cells. These B cells are critically dependent on their interactions with CD4^+^ follicular helper T cells (Tfh) which provide signals necessary for the survival and proliferation of these B cells (24–26).

The individual macrophages for all patients were plotted based on the intensity of CD86, CD163, and CD206 (**Figure 3G**), providing a spectrum of macrophage populations according to MTA1 levels. These data clearly displayed evidence of differential macrophage polarization between the MTA1-high and MTA1-low groups.

### Colorectal Cancer Cells Expressing Higher Levels of MTA1 Induced Macrophage Polarization Into M2-Like Tumor-Associated Macrophage (TAM) Phenotypes

**Figure 4:**
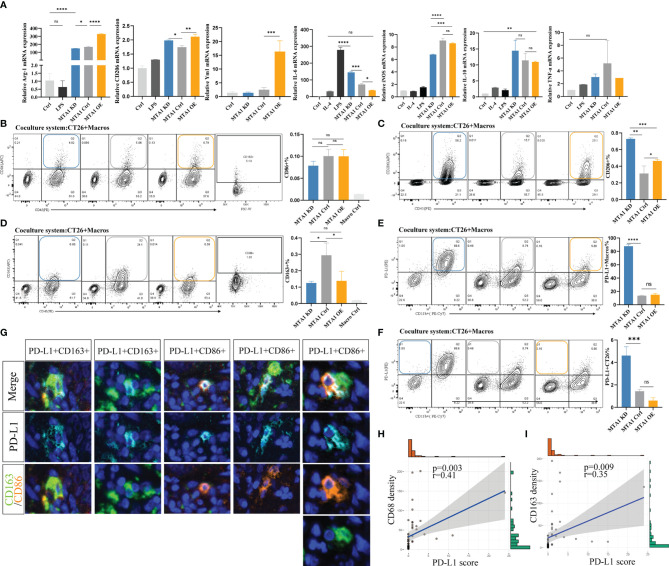
MTA1 drives the polarization of macrophages in colorectal cancer. **(A)** qRT–PCR analysis of macrophage polarization-related genes in cell line Raw264.7 cocultured with cell line CT26 (MTA1-ctrl), and CT26 depleted for MTA1 (MTA1-KD) or enforced to express MTA1(MTA1-OE) for 48 h. **(B)** Flow cytometry analysis of macrophage polarization-related marker CD86 in cell line Raw264.7 cocultured with cell line CT26 of the control (MTA1-ctrl) and CT26 depleted of MTA1 (MTA1-KD) or enforced to express MTA1(MTA1-OE) for 48 h. **(C)** Flow cytometry analysis of macrophage polarization-related marker CD206 in cell line Raw264.7 cocultured with cell line CT26 (MTA1-ctrl), CT26 depleted of MTA1 (MTA1-KD) or CT26-overexpressing MTA1(MTA1-OE) for 48 h. **(D)** Flow cytometry analysis of macrophage polarization-related marker CD163 in cell line Raw264.7 cocultured with cell line CT26 (MTA1-ctrl), CT26 depleted of MTA1 (MTA1-KD) or CT26-overexpressing MTA1 (MTA1-OE) for 48 h. **(E)** Flow cytometry analysis of immune checkpoint, PD-L1, in cell line Raw264.7 cocultured with cell line CT26 (MTA1-ctrl), CT26 depleted of MTA1 (MTA1-KD) or CT26-overexpressing MTA1(MTA1-OE) for 48 h. **(F)** Flow cytometry analysis of immune checkpoint PD-L1 in cell line CT26 (MTA1-ctrl), CT26 depleted of MTA1 (MTA1-KD) or CT26-overexpressing MTA1(MTA1-OE) cocultured with cell line Raw264.7for 48 h. **(G)** The expression pattern of PD-L1 in clinical colorectal cancer tissue array. **(H)** Correlation between PD-L1 score and infiltration density of CD68^+^ macrophages in colorectal cancer tissue array. **(I)** Correlation between PD-L1 score and infiltration density CD163^+^ macrophages in colorectal cancer tissue array. The meaning of symbols was provided in Statistical Analysis.

To explore the influence of MTA1 expression in tumor cells on the polarization of macrophages in the tumor microenvironment, we separately cocultured MTA1-knockdown, MTA1-overexpressing and control CT26 mouse colorectal cancer cells with mouse macrophages. Macrophages cocultured with MTA1-overexpressing colorectal cancer cells significantly upregulated the M2-like macrophage markers Arg-1, CD206 and Ym1 (**Figure 4A**) but significantly downregulated the expression of the immunostimulator IL-6 (**Figure 4A**). The differences in the expression of the M1-like macrophage marker INOS and the immunoinhibitors TNF-α and IL-10 were not significant between the groups (**Figure 4A**) (27). In the results of phenotype analysis by flow cytometry, colorectal cancer cells expressing higher MTA1 promoted the polarization of macrophages to the CD206^+^ phenotype (**Figure 4C**) while inhibiting CD163^+^ polarization (**Figure 4D**) without affecting the polarization of the CD86^+^ phenotype (**Figure 4B**). In addition, we found that CT26 colorectal cancer cells barely expressed PD-L1 (**Figure 4F**). In the coculture system, PD-L1 was mainly expressed on macrophages, and CT26 cells expressing lower MTA1 expression promoted macrophages to express significantly upregulated PD-L1 in comparison with other groups (**Figure 4E**). By staining PD-L1 expression on the cancer tissue array from our clinical colorectal cohort, we confirmed the expression pattern of PD-L1 in colorectal cancer. The expression of PD-L1 in tumor cells was relatively lower than that in macrophages. The expression of PD-L1 was relatively higher in macrophages and other cells that were not stained in our panels (**Figure 4G**). Analysis of the correlation of the PD-L1 immunohistochemical score and the macrophage infiltration density revealed a significant positive/negative correlation between PD-L1 expression and macrophage infiltration density in colorectal cancer (**Figures 4H, I**).

In total, MTA1-overexpressing colorectal cancer cells significantly induced polarization of macrophages into M2 tumor-associated macrophage (TAM) phenotypes. In addition, we found that colorectal cancer cells rarely expressed PD-L1, suggesting that PD-L1 in the colorectal tumor microenvironment was mainly produced by macrophages and other nontumor cells.

### Macrophages Help Kill T Cells in Colorectal Cancer Models

**Figure 5:**
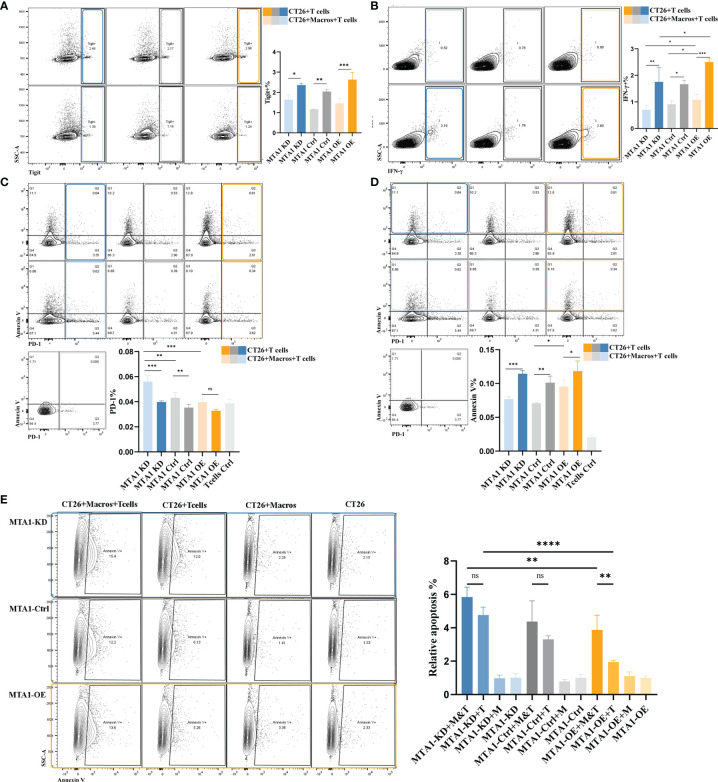
MTA1-driven functional activation of T cells in colorectal cancer. **(A)** Flow cytometry analysis of immune checkpoint Tigit in T cells isolated from mouse spleen and cocultured with cell line CT26 (MTA1-ctrl), CT26 depleted of MTA1 (MTA1-KD) or CT26 overexpressing MTA1(MTA1-OE) for 6 h. **(B)** Flow cytometry analysis of activation marker of T cell, Ifng, in T cells isolated from mouse spleen cocultured with cell line CT26 (MTA1-ctrl), CT26 depleted of MTA1 (MTA1-KD) or CT26 overexpressing MTA1(MTA1-OE) for 6 h. **(C)** Flow cytometry analysis of immune checkpoint PD1 in T cells isolated from mouse spleen cocultured with cell line CT26 (MTA1-ctrl), CT26 depleted of MTA1 (MTA1-KD) or CT26 overexpressing (MTA1-OE) for 6 h. **(D)** Flow cytometry analysis of apoptosis using Annexin V in T cells isolated from mouse spleen cocultured with cell line CT26 ((MTA1-ctrl), CT26 depleted of MTA1 (MTA1-KD) or CT26 overexpressing MTA1(MTA1-OE) for 6 h. **(E)** Flow cytometry analysis of apoptosis marker, Annexin V, in cell line CT26 ((MTA1-ctrl), depleted of MTA1 (MTA1-KD) or overexpression for MTA1(MTA1-OE) cocultured with T cells isolated from mouse spleen for 6 h. (M, Macrophages; T, T cells). The meaning of symbols was provided in Statistical Analysis.

As above, we found a positive correlation between the MTA1 expression level and CD8^+^ T cell infiltration. CD8^+^ T cells are the main immune cells that directly exert antitumor effects. Therefore, we focused on the influence of MTA1 expression and its influence on macrophages on the activation of T cells. The results from flow cytometry showed that higher MTA1 expression in CT26 colorectal cancer cells promoted the secretion of Ifng in T cells (**Figure 5B**), reflecting T cell activation (28). In addition, we detected the immune checkpoints PD-1 and Tigit in T cells (29) and found that the expression of PD-1 (**Figure 5C**) and Tigit (**Figure 5A**) was not significantly different between the groups. However, the presence of macrophages in the coculture system significantly upregulated PD-1 expression levels (**Figure 5C**) and downregulated the expression of Ifng (**Figure 5B**) and Tigit (**Figure 5A**) in T cells. This suggests that macrophages suppress the activation of T cells. However, the presence of macrophages significantly decreased the apoptosis of T cells in the coculture system (**Figure 5D**), especially in the MTA1 knockdown group (**Figure 5D**).

Next, we performed a T cell cytotoxicity assay to further investigate the effect of MTA1 expression in colorectal cancer cells on T cell cytotoxicity in the tumor microenvironment (**Figure 5E**). Macrophages alone did not show a tumor-killing effect, while they enhanced the killing effects of T cells on tumor cells in the coculture system. In the coculture system, cancer cells expressing higher MTA1 suppressed the tumor-killing effect of T cells, and additional macrophages increased the killing effects of T cells on cancers.

These results indicated that MTA1 overexpression-correlated macrophage exclusion in tumor cells exacerbates the immunosuppressive state of the tumor macroenvironment.

### MTA1 Overexpression Attenuated the Interaction Between Cancer Cells and Effector T Cells, Which Could be Rescued by Macrophage Completion

**Figure 6:**
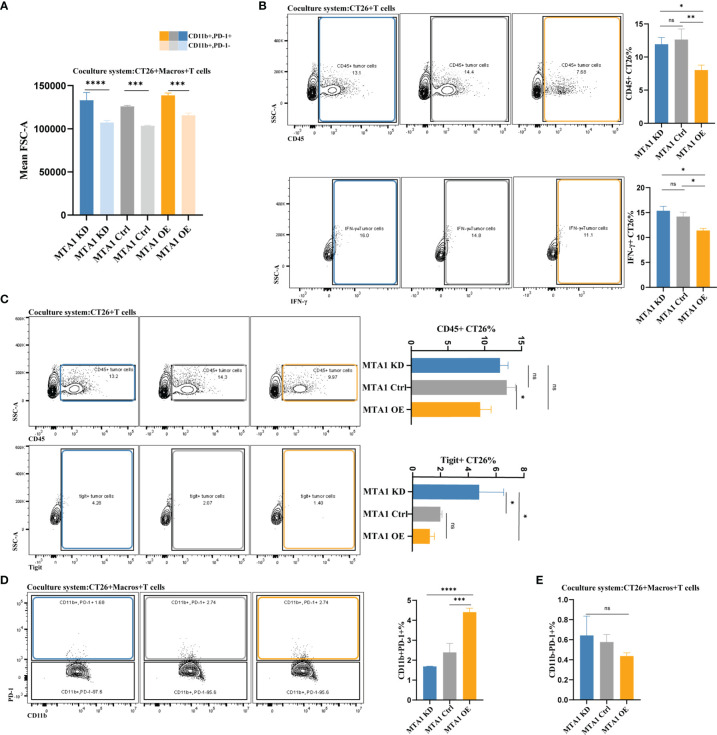
MTA1-driven difference in interaction between tumor cells, macrophages and T cells in colorectal cancer. **(A)** The average volume variation of a single macrophage or object of interacting T cells and macrophages. **(B)** Flow cytometry analysis of the interaction between T cells (all CD45-positive T cells and Tigit-positive T cells) and CT26 tumor cells (MTA1-ctrl), CT26 cells depleted of MTA1 (MTA1-KD) or CT26 cells overexpressing MTA1 (MTA1-OE). **(C)** Flow cytometry analysis of interaction between T cells (all the CD45 positive T cells and Ifng positive T cells) and tumor cells, CT26 (MTA1-ctrl), depleted of MTA1 (MTA1-KD) or overexpression of MTA1 (MTA1-OE). **(D)** Flow cytometry analysis of the interaction between T cells (all the CD45 positive T cells and Tigit positive T cells) and macrophages cocultured with CT26 (MTA1-ctrl), depleted of MTA1 (MTA1-KD) or overexpression for MTA1 (MTA1-OE) for 6 h. **(E)** Flow cytometry analysis of interaction between T cells (PD1 positive T cells) and tumor cells, CT26 (MTA1-ctrl), depleted of MTA1 (MTA1-KD) or overexpression of MTA1 (MTA1-OE) in the cocultured system with macrophages. The meaning of symbols was provided in Statistical Analysis.

The most important manner in which effector T cells kill cancer cells is to directly interact with tumor cells and establish immunological synapses to perform accurately polarized secretion, ensuring that CTLs destroy only the recognized cells but not neighboring bystanders (30). Thus, we analyzed the interaction between tumor cells and T cells under various conditions with or without macrophages. First, we primarily compared the detection index of FCS, which represents the volume of cells, to exclude potential fluorescence crosstalk signals. The average FSC-A was equally increased in the cancer cell-T cell coculture system compared with the single cancer cell groups, indicating the reliability of the system to detect the interaction between T cells and cancer cells (**Figure 6A**). We found that the interaction between tumor cells and T cells in the MTA1-overexpressing group was significantly decreased compared to that of the control and MTA1-knockdown groups. Furthermore, when labeling the T cells with the immune markers Ifng or Tigit, the interaction between tumor cells and both T cells expressing Ifng (**Figure 6B**) or T cells expressing the immune checkpoint Tigit was significantly decreased in the MTA1-overexpressing group (**Figure 6C**). When macrophages were added to the coculture system, the difference in the interaction between different groups became insignificant. In addition, the interaction between macrophages and PD-1^+^ T cells was significantly increased by MTA1 overexpression (**Figure 6D**). However, the addition of macrophages enhanced the interaction between T cells and MTA1-overexpressing tumor cells, eliminating the difference between the interaction of T cells with MTA1-high and MTA1-low cancer cells (**Figure 6E**).

In summary, the high expression of MTA1 in colorectal cancer decreased the interaction between tumor cells and T cells, which can be rescued by the presence of classical macrophages. Unfortunately, with still unverified mechanisms, macrophages were always lacking in the tumor microenvironment with MTA1 overexpression. The combinational consequence of MTA1 overexpression is a markedly suppressed immune microenvironment.

## Discussion

As we recognize tumors as a complex microenvironment in which various cell types interact with each other, contributing to the phenotypes of the entire tumor, we are facing the new challenge of understanding the mechanisms of the interaction network between cell types and how they influence cancer treatment practice (1, 31, 32).

Due to progress in biological technologies, it has been possible to identify different subclasses in the immune microenvironment (TIME) of tumors. The immune signatures in the microenvironment are predictive of patient outcomes and the response to immunotherapy and leads to novel therapeutic strategies (33–35). Therefore, it is urgent to interpret the immune profiles in cancer.

In our previous research, we reported overexpression of MTA1 in the majority of cancer types, which contributes to the malignant phenotypes of cancers, especially metastatic behaviors (36–39). We found clues that highly expressed MTA1 is associated with immune signatures in the tumor microenvironment. Here, we report the ability of MTA1 to rewire the transcriptome and proteome of colorectal cancer cells, resulting in an immunosuppressive microenvironment. Mechanistically, we described that the chemokines and cytokines downregulated by MTA1 in cancer cells may result in decreased macrophages in the tumor microenvironment. We also found that macrophage polarization was affected. Colorectal cancer cells expressing high levels of MTA1 tend to induce M2-like macrophages. The polarization phenotypes were significantly different in MTA1-high versus MTA1-tumors.

In addition, we found enrichment of CD8+ T cells in the MTA1-high patient group. However, the enrichment of CD8^+^ T cells was not significantly correlated with patient survival. We hypothesized that the enriched CD8^+^ T cells were functionally impaired or that cancer cells expressing high levels of MTA1 may be intrinsically resistant to the killing effects of CD8^+^ T cells. Subsequent investigation showed that decreased macrophages at high MTA1 levels significantly affected the tumor killing of T cells. The completion of macrophages into the coculture system significantly restored the tumor-killing function of T cells. The existence of macrophages in the microenvironment significantly improved the interaction between T cells and cancer cells. Numerous studies have also mentioned the essential crosstalk between myeloid cells and T cells in the antitumor immune response (40–43). Activated T cells stimulated by aPD-1 or other cytokines expressed IFN-γ and other proinflammatory cytokines; however, the antitumor effect of activated T cells was dependent on the presence of myeloid cells, such as DCs, CD14^+^ macrophages and CXCL9^+^ macrophages. Not only the presence of myeloid cells but also crosstalk between myeloid cells and T cells were necessary for the antitumor effect of T cells. In our study, we also emphasized that the relatively decreased macrophages impaired the antitumor effect in MTA1-overexpressing colorectal cancer. However, the crosstalk network between myeloid cells and T cells still needs to be solved, and the detailed mechanisms by which myeloid cells stimulate antitumor phenotypes should be further clarified to design clinical therapeutic strategies.

**Figure 7:**
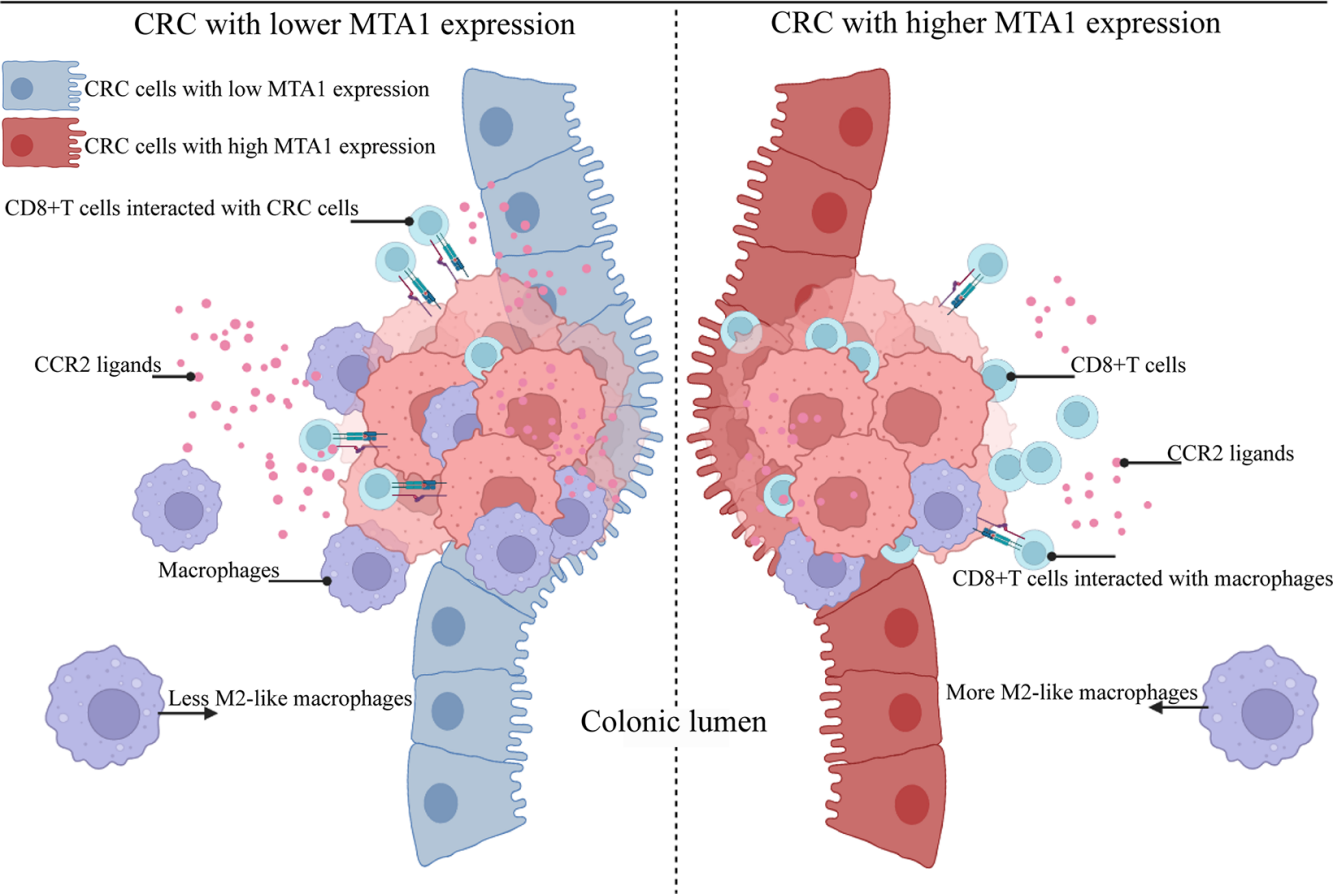
Schematic model of colorectal cancer with lower and higher MTA1 expression. Summary of the main conclusions from this study. In brief, MTA1 was found to rewire the transcriptome and proteome of colorectal cells to promote an immunosuppressive tumor microenvironment. MTA1-overexpressing colon cancer cells significantly decreased the expression levels of the CCR2 ligands CCL2 and CCL7, among others, which were able to attract macrophages. This microenvironment of MTA1-overexpressing colorectal cancer ultimately leads to CD8 T cell enrichment but a lack of classical macrophages. Macrophages enhanced the cytotoxic effect of T cells while enhancing the interaction of T cells and cancer cells. In MTA1-overexpressing colorectal cancer, the absence of macrophages impaired the cytotoxic effect of T cells, decreased the interaction of T cells and cancer cells, and finally resulted in an immunosuppressive tumor microenvironment.

MTA1 was first reported as a metastasis-associated molecule in breast cancer, and then, the functions and tumorigenic potential of MTA1 were gradually recognized. MTA1 affects the biological behaviors of tumors from genetic and epigenetic aspects (36–39). In this study, the results suggested an MTA1-derived immunosuppressive signature in the tumor microenvironment. In more detail, MTA1 remodeled the tumor microenvironment into a CD8^+^ T cell-enriched and classical macrophage-lacking microenvironment (**Figure 7**). Based on the characteristics of the MTA1-derived immunosuppressive microenvironment, we can further focus on enhancing the tumor-killing effect of CD8^+^ T cells by interfering with the MTA1 level since it intermediates the interaction between CD8^+^ T cells and tumor cells.

There are certain limitations to roundly defining the detailed functions of CD8^+^ T cell subtypes and macrophage subtypes induced by MTA1-high tumor cells. In this study, we only focused on the tumor-killing pathway by cytotoxicity. This research suggested that the absence of macrophages significantly decreased the cytotoxicity of CD8^+^ T cells. Limited by the complex interaction and secretion in the tumor microenvironment, we have not yet defined the core chemokine regulation networks between tumor cells, CD8^+^ T cells and macrophages derived by MTA1. According to the results in this study, it is highly probable that macrophages enhance cytotoxicity by maintaining the survival of CD8^+^ T cells and antigen presentation (44), and it is worth elucidating the detailed mechanism in further studies.

In conclusion, our results show that MTA1 overexpression in colon cancer drives a CD8^+^ T cell-rich but exhausted phenotype by decreasing macrophage intensity and inducing M2-like macrophage polarization. The importance of MTA1 on macrophages for the antitumor effects of CD8^+^ T cells implies an immunosuppressive tumor microenvironment by MTA1 overexpression in cancers. Our report guarantees further study on whether MTA1 can serve as a marker for the sensitivity of cancers to immunotherapy or even as an immunotherapy target in combination with current immune checkpoint blockers.

## Data Availability Statement

The datasets presented in this study can be found in online repositories. The names of the repository/repositories and accession number(s) can be found in the article/**Supplementary Material**.

## Ethics Statement

The studies involving human participants were reviewed and approved by the Cancer Hospital, Chinese Academy of Medical Sciences National GCP Center for Anticancer Drug, The Independent Ethics Committee. The patients/participants provided their written informed consent to participate in this study. The animal study was reviewed and approved by the Animal Control Committee of National Cancer Center/National Clinical Research Center for Cancer/Cancer Hospital, Chinese Academy of Medical Sciences and Peking Union Medical College.

## Author Contributions

Conceptualization: HQ, FM, and DX Experiment: YZhou and PN. Data analysis: YZhou, CL, PN, HM, YZhang, and HW. Resources: HQ and DX. Manuscript writing: YZhou. Manuscript editing: HQ. Visualization: YZhou, CL, PN, HM, HW, and YZhang. Supervision: HQ, FM, and DX. Project Administration: HQ. Funding Acquisition: HQ. All authors listed have made a substantial, direct, and intellectual contribution to the work and approved it for publication.

## Funding

This work was financially supported by grants from the National Key Research and Development Program of China (2021YFF1201302), the National Natural Science Foundation of China (No. 81872280, 82073094), the CAMS Innovation Fund for Medical Sciences (CIFMS)(2021-I2M-014), the Open Issue of State Key Laboratory of Molecular Oncology (No. SKL-KF-2021-16), and the Independent Issue of State Key Laboratory of Molecular Oncology (No. SKL-2021-16).

## Conflict of Interest

Author HW was employed by Chinese Medical Journals Publishing House Co., Ltd.

The remaining authors declare that the research was conducted in the absence of any commercial or financial relationships that could be construed as a potential conflict of interest.

## Publisher’s Note

All claims expressed in this article are solely those of the authors and do not necessarily represent those of their affiliated organizations, or those of the publisher, the editors and the reviewers. Any product that may be evaluated in this article, or claim that may be made by its manufacturer, is not guaranteed or endorsed by the publisher.
